# Novel Ultrafine Fibrous Poly(tetrafluoroethylene) Hollow Fiber Membrane Fabricated by Electrospinning

**DOI:** 10.3390/polym10050464

**Published:** 2018-04-24

**Authors:** Qinglin Huang, Yan Huang, Shangpeng Gao, Mengyuan Zhang, Changfa Xiao

**Affiliations:** State Key Laboratory of Separation Membranes and Membrane Processes/National Center for International Joint Research on Separation Membranes, Department of Material Science and Engineering, No. 399 West Binshui Road, Xiqing District, Tianjin Polytechnic University, Tianjin 300387, China; tjpuhuangyan@163.com (Y.H.); tjpugsp@163.com (S.G.); zhang_mengyuan17@163.com (M.Z.); cfxiao@tjpu.edu.cn (C.X.)

**Keywords:** electrospinning, ultrafine fibrous, PTFE, hollow fiber membrane

## Abstract

Novel poly(tetrafluoroethylene) (PTFE) hollow fiber membranes were successfully fabricated by electrospinning, with ultrafine fibrous PTFE membranes as separation layers, while a porous glassfiber braided tube served as the supporting matrix. During this process, PTFE/poly(vinylalcohol) (PVA) ultrafine fibrous membranes were electrospun while covering the porous glassfiber braided tube; then, the nascent PTFE/PVA hollow fiber membrane was obtained. In the following sintering process, the spinning carrier PVA decomposed; meanwhile, the ultrafine fibrous PTFE membrane shrank inward so as to further integrate with the supporting matrix. Therefore, the ultrafine fibrous PTFE membranes had excellent interface bonding strength with the supporting matrix. Moreover, the obtained ultrafine fibrous PTFE hollow fiber membrane exhibited superior performances in terms of strong hydrophobicity (CA > 140°), high porosity (>70%), and sharp pore size distribution. The comprehensive properties indicated that the ultrafine fibrous PTFE hollow fiber membranes could have potentially useful applications in membrane contactors (MC), especially membrane distillation (MD) in harsh water environments.

## 1. Introduction

Poly(tetrafluoroethylene) (PTFE) is a type of high-performance perfluoropolymer with a unique combination of outstanding chemical resistance, excellent thermal stability, strong hydrophobicity, exceedingly good antifouling property, and so on [1]. These exceptional properties make it an ideal material for industrial filtration, wastewater treatment, and membrane contactors (MC), especially for membrane distillation (MD) in harsh water environments (such as strong acid/alkali solution, corrosive solvent, or extreme temperature) [2,3,4].

However, owing to the ultrahigh melt viscosity and its insolubility, PTFE membranes or fibers are difficult to produce by conventional solution-spinning or melt-spinning methods [5,6]. Nowadays, the commercial PTFE membranes are usually prepared through the uniaxial or biaxial stretching method [7,8]. However, it is especially difficult to produce the PTFE hollow fiber membrane, which is favored for its larger effective membrane area, good mechanical property and ease of handling in comparison to the PTFE flat-sheet membrane [9,10,11]. The paste-extrusion method of producing the PTFE hollow fiber membrane requires a high quantity of lubricants in the preparation process, which would result in considerable environmental pollution [11,12,13]. Moreover, the method requires cumbersome and expensive equipment, and produced membranes always have low porosity and limited permeate flux [14,15].

Electrospinning has proven to be an effective, straightforward, convenient, and versatile technology to synthesize continuous submicron structures from a huge range of materials [16,17]. In addition, it can produce highly porous nonwoven filtration media with a high specific surface area and a compact structure [18]. Generally, additives were added into the PTFE emulsion to aid the preparation of the PTFE membrane by the electrostatic spinning method. Xiong et al. [8] reported a groundbreaking work in which fibrous PTFE membranes were fabricated by blending poly(vinylalcohol) (PVA) into a PTFE emulsion through electrospinning and sintering, and Chuyang Y. Tanga et al. [19] evaluated their performance in the application of oil–water separation. Until now, there have been few studies about the fabrication of the PTFE hollow fiber membrane by the electrospinning method.

In our previous work, we fabricated ultrafine fibrous PTFE flat-sheet membranes by sintering electrospun PTFE/poly(vinylalcohol) (PVA) ultrafine fibrous membranes, and their performance in the application of vacuum membrane distillation (VMD) was investigated [20,21]. Based on our previous work, we reported a novel method of fabricating ultrafine fibrous PTFE hollow fiber membranes. Specifically, nascent PTFE/PVA ultrafine fibrous membranes were electrospun while covering the outer surface of the glassfiber braided tube, which served as the supporting matrix of the PTFE hollow fiber membrane. During the following sintering process, the spinning carrier PVA was removed while the obtained ultrafine fibrous PTFE membrane shrank inward so as to further integrate with the supporting matrix. Therefore, the ultrafine fibrous PTFE membranes have excellent interface bonding strength with the supporting matrix. The performances of the PTFE hollow fiber membrane in terms of hydrophobicity, porosity, permeability, and so on were investigated respectively.

## 2. Materials and Methods

### 2.1. Materials

PTFE aqueous dispersion (FR301B) was purchased from 3F New Materials Co., Ltd., Shanghai, China. The specifications of the dispersion are shown in Table S1 (Supplementary material). PVA (Type1788, degree of polymerization 1700, and degree of alcoholysis 87.0–89.0%) was purchased from Aladdin Industrial Co., Ltd., Shanghai, China. Poly(acrylonitrile) (PAN, P1361) was purchased from Spectrum Chemical Mfg. Corp., Gardena, CA, USA. Boric acid was obtained from Yingda Rare Chemical Reagents Factory, Tianjin, China. *N*,*N*-dimethylformamide (DMF) was purchased from Tianjin Kermel Chemical Reagents Co., Ltd., Tianjin, China. All the reagents were used as purchased without further purification. A glassfiber braided tube was provided by Dongguan Goddess Co., Ltd., Guangdong, China, and the basic properties of the glassfiber braided tube are shown in Figure S1.

### 2.2. Membrane Preparation

PVA aqueous solution (10 wt%) and PAN solution (10 wt%) were prepared by dissolving the materials in distilled water and DMF respectively. Then, PTFE emulsion and a certain amount of boric acid (0 wt% and 0.025 wt% of the solution) were added into PVA solution to obtain a homogeneous spinning solution. The composition of the spinning solution was tabulated in Table S2. The spinning solutions were named S-1 and S-2 respectively, and the corresponding membranes were named M-1 and M-2 respectively. The M-3 membrane was fabricated by electrospinning S-2 on a glassfiber braided tube, and then electrospun with S-1 covering on S-2. The glassfiber braided tube was pretreated before its use with solvent treatment and heat treatment to remove the agent on the fiber surface.

**Figure 1:**
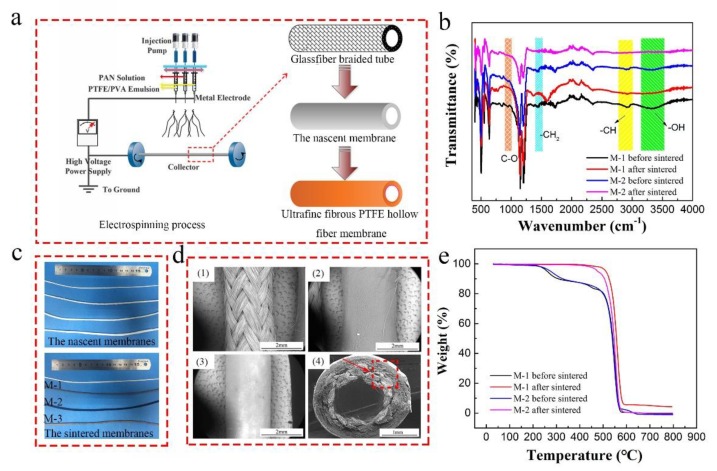
(**a**) Schematic diagram of the preparation process; (**b**) Fourier-transform infrared (FTIR) spectra of prepared membranes; (**c**) Photos of nascent and sintered hollow fiber membranes; (**d**) SEM images of (1) porous glassfiber braided tube; (2) the nascent membrane; (3) the sintered membrane; (4) the whole cross-section of the sintered membrane; (**e**) Thermogravimetric (TG) curves of prepared membranes.

The schematic of electrospinning PTFE hollow fiber membranes was shown in Figure 1. The spinning parameters of electrospinning PTFE/PVA ultrafine fibrous membranes were tabulated in Table 1. In summary, the glassfiber braided tube fixed on conductive wire served as the collector as well as the inner layer, while the electrospun submicron fibers covered on the supporting tube serves as the outer layer of of the hollow fiber membranes. During the electrospinning process, a negative and positive voltage was applied on the conductive wire and needle respectively, and the submicron fibers were assembled on the supporting tube under electrostatic attraction. Co-electrospun PAN nanofibers were utilized to reinforce the electrospun ultrafine fibrous PTFE membranes. As for the precursor composite PTFE/PVA nanofiber membrane, the mechanical strength and support performance was relatively weak. Therefore, the co-electrospun PAN nanofibers were introduced to reinforce the precursor composite membrane because of their good mechanical strength and support performance. The nascent PTFE/PVA membrane was dried for at least 8 h at 70 °C and sintered to 380 °C for 10 min at a heating rate of 1 °C/min in air atmosphere. During the sintering process, PVA in the nascent membrane decomposed, while the sintered ultrafine fibrous PTFE hollow fiber membrane obtained covering on the glassfiber braided tube

### 2.3. Characterization

#### 2.3.1. Membrane Morphologies

The membrane morphologies were investigated by scanning electron microscopy (FESEM S4800 and SEM TM3030, Hitachi, Tokyo, Japan) after coating the membrane with gold. The membrane surface roughness was investigated by confocal laser scanning microscope (Zeiss CSM700, Zeiss, Gttingen, Germany). The diameter distribution of the ultrafine fibers was calculated from the SEM images using Image Proplus software.

#### 2.3.2. Structure Analyses

Fourier-transform infrared spectroscopy (FTIR, Nicolet iS5, iD7 ATR, Thermo Fisher Scientific, Madison, WI, USA) was used to identify the surface chemistry composition of the ultrafine fibrous membranes before and after sintering. The crystalline phases of the samples were confirmed by X-ray diffraction (XRD, D8 Discover, Bruker, Karlsruhe, Germany), with the diffraction angle 2θ having a value from 10° to 45°. The standard thermogravimetric (TG) analysis was completed using a thermogravimetric analysis instrument (TA-SDT Q600, TA instruments, New Castle, DE, USA) (air atmosphere, heating rate: 5 °C/min, 25–800 °C). 

#### 2.3.3. Membrane Properties

Water contact angle (WCA) measurements were performed using an optical contact angle meter (model DSA100, KRUSS Co., Hamburg, Germany) by the sessile drop method of water drops, and more than fifteen readings were obtained for each sample. The mean pore size and porosity were tested using an automatic mercury porosimeter (Auto pore IV9500, Micromeritics, Norcross, GA, USA). The membranes’ liquid entrance pressure (LEP) was determined using a self-made setup which is illustrated in Figure S2.

## 3. Results and Discussion

### 3.1. Formation mechanism

The FTIR spectra of the ultrafine fibrous PTFE hollow fiber membranes before and after sintering are shown in Figure 1b. All the spectra showed obvious peaks at 1250 cm^−1^ (C–F) and 1150 cm^−1^ (C–C), which corresponded to the stretching vibrations of PTFE. For the spectrum of nascent PTFE membranes (Figure 1b), the absorption peaks at 3330 cm^−1^ (O–H), 2910 cm^−1^ (C–H), 1460 cm^−1^ (CH_2_), and 952 cm^−1^ (C–O) were assigned to PVA [22]. These peaks gradually disappeared during the sintering process, which confirmed that the PVA decomposed completely while the PTFE remained. These were consistent with the results of TG analysis (Figure 1e). In addition, there were two decreases in weight in the TG curves, which were attributed to the decomposition of PVA (from 220 °C to 350 °C) and PTFE (from 425 °C to 580 °C) respectively. There was almost no weight change between 335 °C and 425 °C, indicating that the PVA decomposed completely, while the PTFE maintained stability. Moreover, the co-electrospun PAN nanofibers showed a typical nitrile (C≡N) peak at 2245 cm^−1^.

### 3.2. Morphology and structure

Owing to the non-self-supporting property of flat-sheet micro-nano fibrous mats, they must be coupled with additional porous backing materials. The porous glassfiber braided tubes are mainly utilized as the backing material of electrical insulation, owing to their excellent mechanical strength, flexibility, and thermal stability [23].

In this study, the porous glassfiber braided tube was used as the supporting matrix of ultrafine fibrous PTFE hollow fiber membranes, which provided not only high mechanical strength but also pressure resistance. As shown in Figure 1c, the electrospun fibers covering the supporting tube serves as the outer layer of the hollow fiber membrane. Figure 1d shows the PTFE ultrafine fibrous layer and the supporting matrix in SEM images. It was found that the membrane showed a good hollow fiber structure and the ultrafine fibrous PTFE layer covered the supporting tube completely. Compared with other studies on nanofiber-covered hollow fiber membranes, the ultrafine fibrous PTFE hollow fiber membrane had much better interfacial bonding between the outer layer and the supporting matrix as the outer layer shrunk inward after the sintering process [15,23].

**Figure 2:**
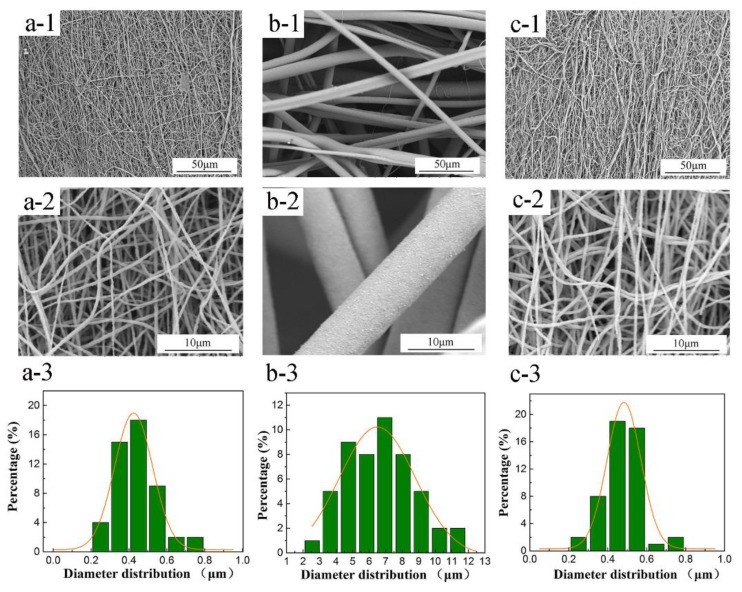
SEM images and the corresponding histograms of fiber diameter distribution of the nascent PTFE/PVA hollow fiber membranes. (**a**) The nascent M-1 membrane; (**b**) the nascent M-2 membrane; (**c**) the nascent M-3 membrane; (1) surface × 800; (2) surface × 5000; (3) fiber diameter distribution.

The morphology of the membranes can provide an insight into the fiber stacking and the fiber’s microstructure as well as its pore network. As shown in Figure 2, the nascent PTFE/PVA hollow fiber membrane exhibited a relatively smooth surface and a uniform fiber diameter. When the boric acid was introduced into the PTFE/PVA spinning solution, the nascent fibers’ diameter of the M-2 membrane clearly increased, and the pore structure became looser compared to the nascent M-1 membrane. These results were attributed to the sol–gel reaction between boric acid and PVA, in which the -OH in PVA reacts with the -H in boric acid, leading to a kind of two-dimensional complex compound which further leads to a higher viscosity of spinning solution. The molecular state and the gelation behavior of the spinning solutions of M-1 and M-2 membranes could be described by rheological behavior, as shown in Figure S3. The shear viscosity increased because of the complex formation between PVA chains that was responsible for the increase in the fibers’ diameters.

**Figure 3:**
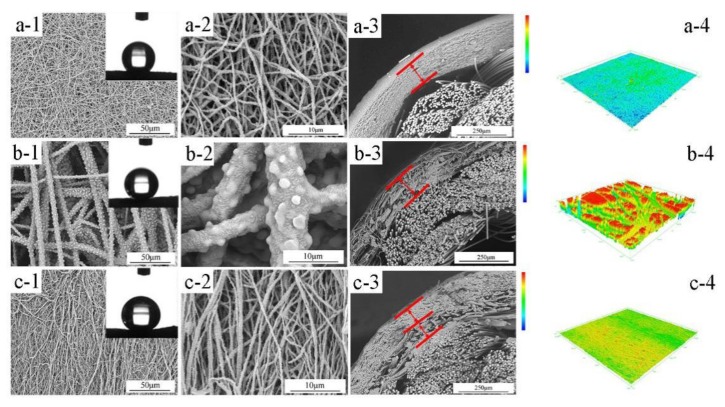
SEM images and the corresponding 3D confocal laser scanning microscope (CLSM) images of the sintered ultrafine fibrous PTFE hollow fiber membranes: (**a**) the sintered M-1 membrane; (**b**) the sintered M-2 membrane; (**c**) the sintered M-3 membrane; (1) surface × 800; insets were the photos of WCA; (2) surface × 5000; (3) the cross-section; (4) 3D CLSM images.

**Figure 4:**
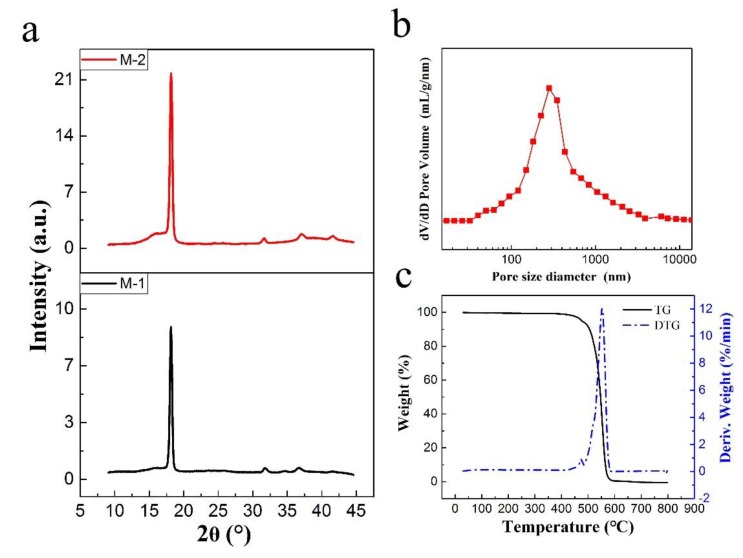
(**a**) XRD patterns of the sintered PTFE hollow fiber membranes, (**b**) Pore size distribution, and (**c**) TG and derivative thermogravimetry (DTG) curves of the sintered M-3 membrane.

After the sintering process, as shown in Figure 3, the highly interconnected porous ultrafine fibrous network was formed. The cross-section morphologies of the prepared membranes showed that there was an obvious effect of boric acid on the morphology of the outer layer. Compared with M-1, M-2 showed a porous outer layer and a better interfacial bonding state because of the enhancement of the spinning solution viscosity. However, it can be seen from Figure 3(b-3,b-4) that the outer layer became looser and rougher, which was consistent with the results of mean pore size and LEPw values (Table 2).

The XRD curves of PTFE hollow fiber membranes are shown in Figure 4a. In the patterns, the PTFE membrane presented a sharp peak at 2θ = 18°, which corresponds to the diffraction from (100) planes in semicrystalline PTFE composed of lamellar crystals which could be indexed to the typical peak of PTFE (JCPDS #47-2217). Also, there was no obvious deviation in the position of the diffraction peak, which indicated that the PTFE crystal type did not change with the addition of boric acid. However, the full width at the half-maximum of the peaks at 2θ = 18° of the sintered M-1 and M-2 membranes were 0.402° and 0.389°, respectively. The X-ray diffraction peak became narrower in conjunction with the increase in X-ray diffraction intensity, which revealed that there was improvement of the crystallinity.

### 3.3. Properties

In general, the permeability increased with an increase in membrane pore size and porosity, while it decreased with the membrane thickness and pore tortuosity porosity. However, it is well known that for a good MD process, the porous membrane should exhibit a high LEPw, and applying a transmembrane hydrostatic pressure that exceeds the LEPw value may lead to pore wetting and a lower quality of the permeate water. The LEPw achieved by a membrane with high hydrophobicity (high water contact angles) and small pore size (ranging from 100 nm to 1 μm) was an essential condition for the process to successfully reduce pore wetting.

The nascent PTFE/PVA membrane was completely wetted by water, which was attributed to the highly hydrophilic PVA. However, the obtained ultrafine fibrous PTFE hollow fiber membrane exhibited strong hydrophobicity (higher than 140°), which were not only owing to the natural hydrophobicity of PTFE, but also the micro-nano structure on the fiber’s surface, which further resulted in a higher membrane roughness. The membrane showed a good hollow fiber structure, and the ultrafine fibrous PTFE layer covered the supporting tube. The hollow porous glassfiber braided tubes increased the resistance of the membrane and provided a high specific surface area. The interaction between nanofibers and rope and the formation of nanofiber layers on the cylindrical rope enhanced the hydrodynamic conditions. Electrospun ultrafine fibrous PTFE fibers served as the outer layer which offered very high porosity, controllable pore size, and strong hydrophobicity. This was undoubtedly beneficial to the MD process.

The porosity and pore size distribution in the results of Table 2 showed that electrospinning could provide a uniform pore size distribution, high interconnectivity of pores, and porosity [24]. It was found that all the prepared membranes exhibited porosity (the entire membrane includes the supporting matrix and the outer layer) values above 62%, and the mean pore size of the M-3 membrane became smaller than the M-2 membrane. Moreover, Figure 4b shows that the M-3 membrane had a narrow pore size distribution, with a mean pore size of 548 nm.

In regards to the LEPw valve, the M-3 membrane held an LEPw value more than 0.2 MPa, which was higher than that of the M-2 membrane. Again, this is due to the larger pore size of the M-2 membrane, although the advancing WCA of the M-2 membrane was higher than that of the M-3 membrane. Moreover, the MD membranes must have good thermal stability and excellent chemical resistance to feed streams. The TG and DTG curves of M-3 membrane shown in Figure 4c also indicated that there was almost no weight change under 400 °C, showing that the ultrafine fibrous PTFE hollow fiber membrane has outstanding thermal stability. Moreover, the porous glassfiber braided tube used as the supporting matrix of the ultrafine fibrous PTFE hollow fiber membrane provided not only high mechanical strength but also pressure resistance for long-term running in the MD process, especially the VMD process.

These results suggested that the ultrafine fibrous PTFE hollow fiber membranes may provide a wide range of potential applications for the wastewater treatment, especially in harsh water environments.

## 4. Conclusions

In summary, we reported a novel method of preparing ultrafine fibrous PTFE hollow fiber membranes by the electrospinning method, with PTFE ultrafine fibrous membranes acting as separation layers while the porous glassfiber braided tube acted as the supporting matrix. The obtained ultrafine fibrous PTFE membranes have excellent interface bonding strength with the supporting matrix. Moreover, ultrafine fibrous PTFE hollow fiber membranes exhibited superior properties such as strong hydrophobicity, high porosity, and sharp pore size distribution. It is expected that the ultrafine fibrous PTFE hollow fiber membrane will attract more attention not only in applications of membrane contactors but also in wastewater treatment.

## Figures and Tables

**Table 1:** The parameters of the nascent poly(tetrafluoroethylene)/poly(vinylalcohol) (PTFE/PVA) hollow fiber membranes.

Characteristic	Value
Voltage (kV)	25 ± 0.1
Relative humidity (%)	70 ± 5
Temperature (°C)	25 ± 3
Spinning distance (cm)	8 ± 0.5
Fluid flow rate (mL/min)	0.008
Collector diameter (mm)	1.5
Collector speed (rpm)	400

**Table 2:** The properties of the sintered PTFE hollow fiber membranes.

Samples	Average Roughness (μm)	WCA (°)	Porosity (%)	LEPw (MPa)	Mean Pore Size (nm)
M-1	0.534	136.4 ± 1.7	62.9	0.24 ± 0.02	555
M-2	5.675	140.2 ± 0.9	73.6	<0.02	1302
M-3	0.615	135.2 ± 1.1	68.7	0.20 ± 0.02	548

## Supplementary Materials

The following are available online at http://www.mdpi.com/2073-4360/10/5/464/s1.
