# Supplementary material for: Novel Ultrafine Fibrous Poly(tetrafluoroethylene) Hollow Fiber Membrane Fabricated by Electrospinning
Source: Polymers (Basel). 2018 Apr 24;10(5):464. doi: 10.3390/polym10050464 (PMC6415508; doi:10.3390/polym10050464)
Supplement: Supplementary file 1 [file polymers-10-00464-s001.pdf]

Supporting Information for

Novel ultrafine fibrous poly(tetrafluoroethylene) hollow fiber membrane by electrospinning

Qinglin Huang*, Yan Huang, Shangpeng Gao, Mengyuan Zhang, Changfa Xiao

State Key Laboratory of Separation Membranes and Membrane Processes/ National Center for International Joint Research on Separation Membranes, Department of Material Science and Engineering, Tianjin Polytechnic University, Tianjin, 300387, P.R. China.

*Corresponding author. E-mail: huangqinglin@tjpu.edu.cn; Tel.: 86-22-83955795, 399 West Binshui Road, Xi Qing District, Tianjin Polytechnic University, Tianjin, 300387, P.R. China.

S1 Characteristics of the PTFE aqueous dispersion 2
S2 The basic properties of glassfiber braided tube 3
S3 The composition of the spinning solutions 4
S4 LEP test 5
S5 Rheological properties 6

S1 Characteristics of the PTFE aqueous dispersion

Table S1. Characteristics of the PTFE aqueous dispersion

Characteristic	Value
Solid Content (wt %)	60
Nonionic Surfactant Content (wt %)	5
Average Particle Size (μm)	0.19
Viscosity (Pa·s)	25×10^{-3}
Density (g/cm^3)	2.20
pH (-)	9

S2 The basic properties of glassfiber braided tube

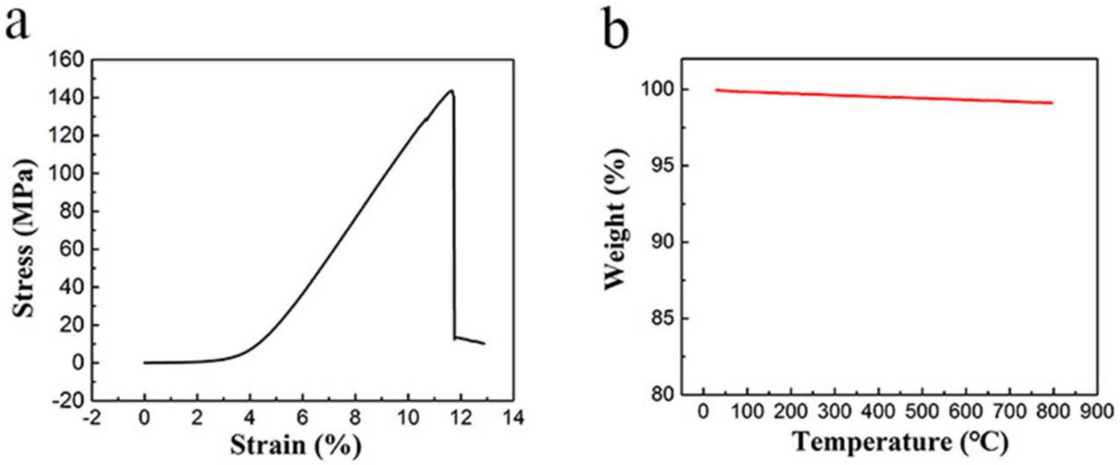

Figure. S1 (a) Stress–strain curve and (b) TG curve of glassfiber braided tube

S3 The composition of the spinning solutions

Table S2. Composition of spinning solution.

Membrane code	Spinning solution Code	PTFE/PVA (wt % / wt %)	The addition of BA (wt % of the solution)
M-1	S-1	6:1	0
M-2	S-2	6:1	0.025
M-3	S-2 were electrospun covering the glassfiber braided tube and then S-1 were electrospun covering S-2		

S4 LEP test

The LEP of water (LEPw) is the minimum pressure required for water to pass through the pores of a dry membrane. During the MD process, the exceeded transmembrane hydrostatic pressure of the LEPw would lead to pore wetting and higher conductivity of the permeate water. The LEPw of membrane was determined using a self-made setup shown in **Figure. S2**.

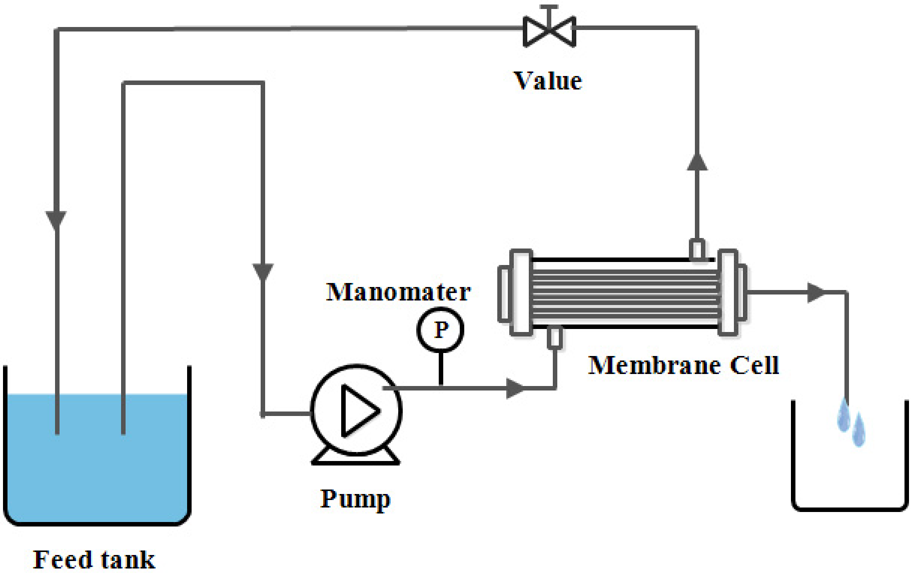

Figure. S2 Schematic diagram of the LEPw testing device

S5 Rheological properties

The rheological behaviour of the spinning solutions was investigated with a rheometer (HAAKE MARS, Thermo Fisher Scientific,Germany). Shear stress was measured over a range of shear rates (γ) from 1 to 100 s^{-1} for each sample. The temperature of spinning solution was 25 ℃. The rheological curves were shown in **Figure. S3.**

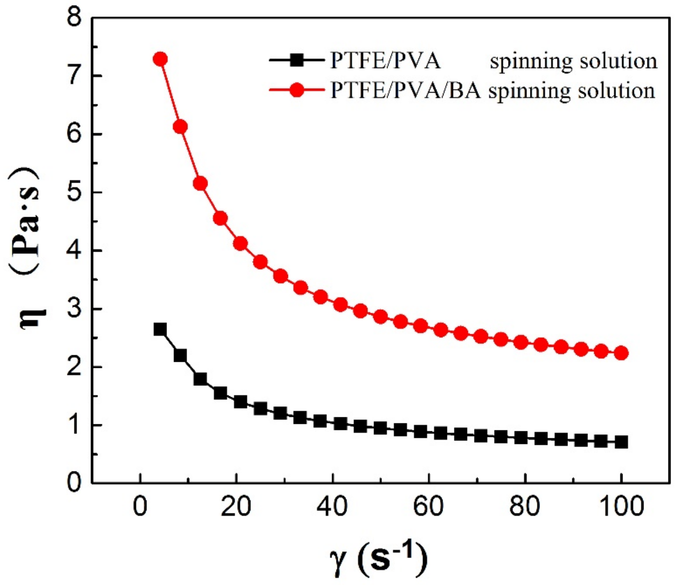

Figure. S3 Rheological curves of different spinning solutions
